# Supplementary material for: Recurrent Duplication and Diversification of Acrosomal Fertilization Proteins in Abalone
Source: Front Cell Dev Biol. 2022 Apr 7;10:795273. doi: 10.3389/fcell.2022.795273 (PMC9022041; doi:10.3389/fcell.2022.795273)
Supplement: Supplementary file 2 [file Table1.doc]

| **Species Name** | **Data Type** | **Citation** | **Geographic Region** |
| --- | --- | --- | --- |
| Haliotis rufescens | Genome | Masonbrink et al 2019 | North America |
| Haliotis rufescens | Testes illumina transcriptome | Palmer et al 2013 | North America |
| Haliotis sorenseni | Genome | <https://abalone.dbgenome.org/downloads> | North America |
| Haliotis fulgens | Genome | <https://abalone.dbgenome.org/downloads> | North America |
| Haliotis discus | Genome | Nam et al 2017 | North America |
| Haliotis rubra | Genome | Gan et al 2019 | Australia |
| Haliotis laevigata | Genome | Botwright 2019 | Australia |
| Haliotis tuberculata | Testes and Ovary PacBio Sequences | Carlisle JA et al 2021 (Present Manuscript) | Europe |

**Supplementary Table 1: Summary of Data Resources Used in Molecular Evolutionary Analysis.**
