# Supplementary figures and images for: Recurrent Duplication and Diversification of Acrosomal Fertilization Proteins in Abalone
Source: Front Cell Dev Biol. 2022 Apr 7;10:795273. doi: 10.3389/fcell.2022.795273 (PMC9022041; doi:10.3389/fcell.2022.795273)

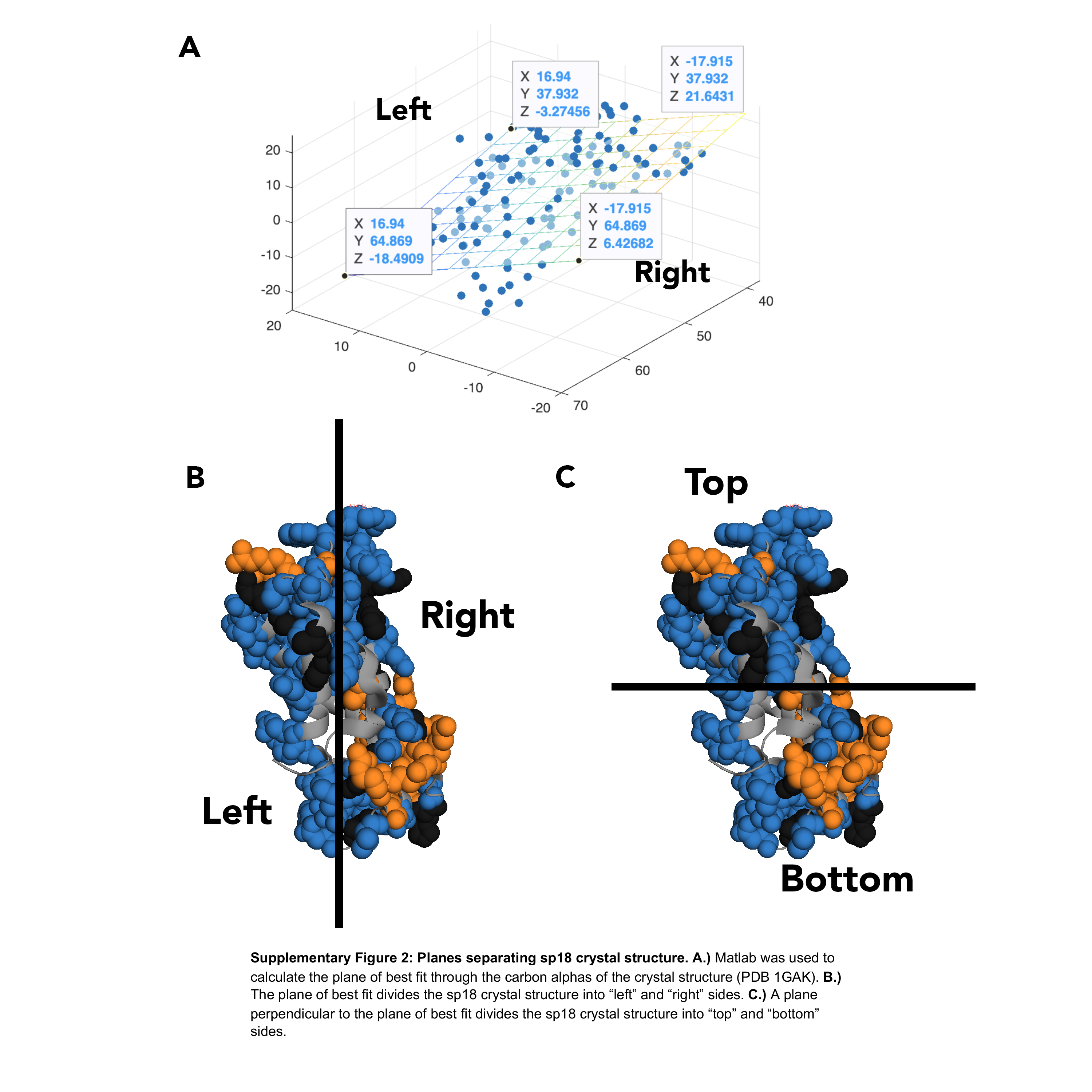

Supplement: Supplementary file 3 [file Image2.PNG]

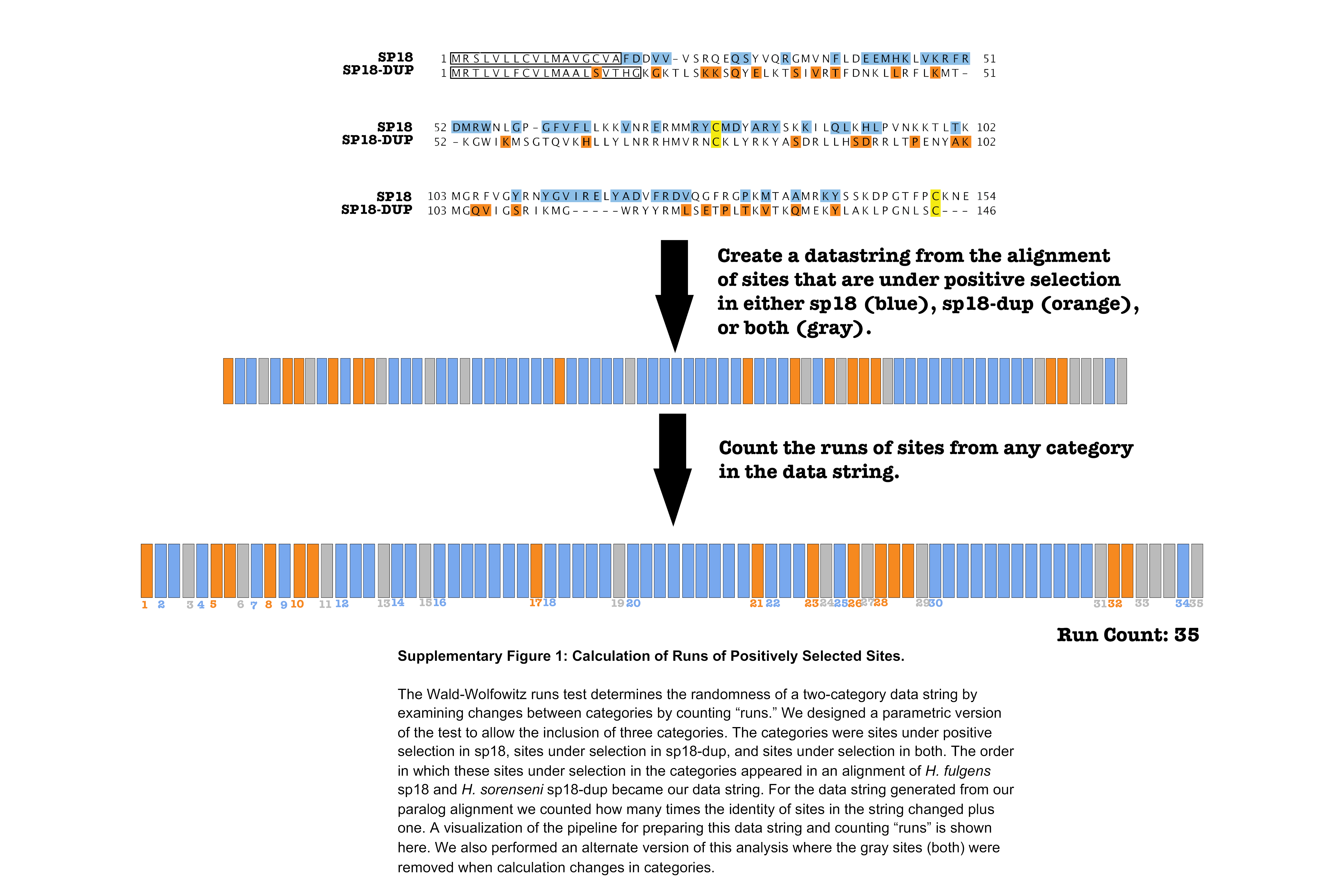

Supplement: Supplementary file 4 [file Image1.PNG]
