# Supplementary material for: Recurrent Duplication and Diversification of Acrosomal Fertilization Proteins in Abalone
Source: Front Cell Dev Biol. 2022 Apr 7;10:795273. doi: 10.3389/fcell.2022.795273 (PMC9022041; doi:10.3389/fcell.2022.795273)
Supplement: Supplementary file 5 [file Table2.doc]

| **Species Name** | **Gene Name** | **GenBank Accession if Available** | **Source** |
| --- | --- | --- | --- |
| H. tuberculata | lysin #1 | OK491874 | Testes Transcriptome PacBio Sequencing |
| H. tuberculata | lysin #2 | OK491875 | Testes Transcriptome PacBio Sequencing |
| H. pustulata | lysin | L35180 | targeted cDNA seq |
| H. midae | lysin | L26275 | targeted cDNA seq |
| H. scalaris | lysin | L26278 | targeted cDNA seq |
| H. laevigata | lysin | L26274 | targeted cDNA seq |
| H. roei | lysin | M98874 | targeted cDNA seq |
| H. cyclobates | lysin | L26271 | targeted cDNA seq |
| H. conicopora | lysin | L26281 | targeted cDNA seq |
| H. rubra | lysin | L26277 | targeted cDNA seq |
| H. diversicolor | lysin | L26272 | targeted cDNA seq |
| H. asinina | lysin | HM582239 | targeted cDNA seq |
| H. iris | lysin | L26273 | targeted cDNA seq |
| H. ovina | lysin | L26276 | targeted cDNA seq |
| H. varia | lysin | L35181 | targeted cDNA seq |
| H. australis | lysin | L26270 | targeted cDNA seq |
| H. fulgens | lysin | L36589 | targeted cDNA seq |
| H. cracherodi | lysin | M59971 | targeted cDNA seq |
| H. corrugata | lysin | M34389 | targeted cDNA seq |
| H. kamtschatkana | lysin | M59970 | targeted cDNA seq |
| H. rufescens | lysin | M34388 | targeted cDNA seq |
| H. sorenseni | lysin | M59968 | targeted cDNA seq |
| H. walallensis | lysin | M59969 | targeted cDNA seq |
| H. discus | lysin | M98875 | targeted cDNA seq |
| H. gigantea | lysin | L26279 | targeted cDNA seq |
| H. rubra | lysin-dup | N/A | Predicted from Genome |
| H. laevigata | lysin-dup | N/A | Predicted from Genome |
| H. rufescens | lysin-dup | N/A | Predicted from Genome |
| H. sorenseni | lysin-dup | N/A | Predicted from Genome |
| H. discus | lysin-dup | N/A | Predicted from Genome |
| H. tuberculata | sp18 # 1 | OK491876 | Testes Transcriptome PacBio Sequencing |
| H. tuberculata | sp18 # 2 | OK491877 | Testes Transcriptome PacBio Sequencing |
| H. rubra | sp18 | N/A | Predicted from Genome |
| H. laevigata | sp18 | N/A | Predicted from Genome |
| H. rufescens | sp18 | L36552 | targeted cDNA seq |
| H. wallalensis | sp18 | MN102340 | targeted cDNA seq |
| H. sorenseni | sp18 | L36553 | targeted cDNA seq |
| H. kamtschatkana | sp18 | L36554 | targeted cDNA seq |
| H. discus | sp18 | MN102341 | targeted cDNA seq |
| H. corrugata | sp18 | L36590 | targeted cDNA seq |
| H. cracherodii | sp18 | MN102342 | targeted cDNA seq |
| H. fulgens | sp18 | L36589 | targeted cDNA seq |
| H. sorenseni | sp18-dup | N/A | Predicted from Genome |
| H. rufescens | sp18-dup | N/A | Testes Transcriptome illumina Sequencing |
| H. discus | sp18-dup | N/A | Predicted from Genome |
| H. laevigata | sp18-dup | N/A | Predicted from Genome |
| H. rubra | sp18-dup | N/A | Predicted from Genome |

**Supplementary Table 2: Summary of lysin, lysin-dup, sp18, and sp18-dup sequences included in phylogenetic analysis and their sources.**
